# Preferences and beliefs in ingroup favoritism

**DOI:** 10.3389/fnbeh.2015.00015

**Published:** 2015-02-13

**Authors:** Jim A. C. Everett, Nadira S. Faber, Molly Crockett

**Affiliations:** ^1^Department of Experimental Psychology, University of OxfordOxford, UK; ^2^Oxford Martin School, University of OxfordOxford, UK

**Keywords:** ingroup favoritism, parochial altruism, prosocial behavior, group processes, behavioral economics

## Abstract

Ingroup favoritism—the tendency to favor members of one’s own group over those in other groups—is well documented, but the mechanisms driving this behavior are not well understood. In particular, it is unclear to what extent ingroup favoritism is driven by preferences concerning the welfare of ingroup over outgroup members, vs. beliefs about the behavior of ingroup and outgroup members. In this review we analyze research on ingroup favoritism in economic games, identifying key gaps in the literature and providing suggestions on how future work can incorporate these insights to shed further light on when, why, and how ingroup favoritism occurs. In doing so, we demonstrate how social psychological theory and research can be integrated with findings from behavioral economics, providing new theoretical and methodological directions for future research.

Across many different contexts, people act more prosocially towards members of their own group relative to those outside their group. Consequently, a number of scientific disciplines concerned with human cognition and behavior have sought to explain such *ingroup favoritism* (also known as *parochial altruism*). Here we explore to what extent ingroup favoritism is driven by *preferences* concerning the welfare of ingroup over outgroup members, vs. *beliefs* about the (future) behavior of ingroup and outgroup members.

In this theoretical review we combine insights from a behavioral economic approach with knowledge from social psychological research on social identity processes in intergroup behavior to explain the proximate psychological causes of ingroup favoritism. We expand upon previous discussions about ingroup favoritism by using a conceptual framework of preferences and beliefs to review present findings demonstrating ingroup favoritism in economic games. Although we focus on economic games here, we also selectively draw upon other related research to highlight how social-psychological theory and research can be incorporated with findings from behavioral economics to provide exciting new directions for research. We therefore provide an integrative review of ingroup favoritism in economic games, identifying key gaps in the literature, as well as providing suggestions on how future work can incorporate these insights to shed further light on when, why, and how ingroup favoritism occurs.

## Social identity and group behavior

From the dawn of our species to the present day, humans have lived, eaten, worked, and reproduced—that is, survived—in groups. These groups have expanded from small, primarily kin-based ties to groups based on language, nationality, religion, current geographical location, and even seemingly arbitrary characteristics such as the ownership of a particular brand of electronic device. As a species, we appear to have a remarkable tendency to seek out and identify with groups, and it has been suggested that cooperation with the ingroup and competition with the outgroup may have co-evolved (c.f. Rusch, 2014). Indeed, it is in our group-based character that the angels and demons of human nature can be seen: on the one hand, the success of intragroup cooperation that has given us democracy and civil rights; and on the other hand, the darkness of intergroup conflict that has given us the collective stains on human history of genocide and war.

The concept of *social identity* (Tajfel, 1970, 1974, 1982) is key to this review—and more broadly most contemporary social psychological work on intergroup processes. Social identity is “that part of an individual’s self concept which derives from his knowledge of his membership of a social group (or groups) together with the value and emotional significance attached to that membership” (Tajfel, 1974, p. 69). We use here the definition of a group from work on intergroup relations in social psychology: a social group is a collection of individuals who perceive themselves to be members of the same social category, and therefore share a social identity (Tajfel and Turner, 1979; Turner et al., 1987; Ellemers et al., 2002; Ellemers and Haslam, 2011; Turner and Reynolds, 2011). Social groups can be based on a range of objective and subjective criteria—from ethnic background to gender to nationality to occupation to religion. An intergroup context emerges when social identities are salient and individuals interact with one another in terms of these social group identities (Turner et al., 1987). Indeed, even assignment to random groups can be sufficient to engender a relevant intergroup context in which intergroup behavior is observed (Tajfel, 1974). Once groups have been formed, how does this influence behavior?

A number of theories have been posited to explain intergroup behavior, including but not limited to the social identity approach (Tajfel and Turner, 1979; Turner et al., 1987); the social exchange and reciprocity model (Yamagishi et al., 1999; Yamagishi and Mifune, 2008); coalitional and tribal instinct-based models (Van Vugt and Schaller, 2008; Van Vugt and Park, 2010); uncertainty reduction theory (Hogg and Abrams, 1993; Hogg, 2000); social dominance theory (Pratto, 1999; Sidanius and Pratto, 1999); and various evolutionary models (e.g., Choi and Bowles, 2007; Fu et al., 2012). With regard to ingroup favoritism specifically, two accounts dominate. On the one hand is the largely preference-based social identity approach generally favored in traditional social psychology (Tajfel and Turner, 1979; Turner et al., 1987), while on the other is a largely belief-based theory of bounded generalized reciprocity (BGR) generally favored by behavioral economists (Yamagishi et al., 1999; Yamagishi and Kiyonari, 2000). Given its prominence in social psychology—and relatively low profile in behavioral economics—we begin by providing a brief overview of the key tenets of the social identity approach to intergroup processes, while also directing the interested reader to existing comprehensive reviews of the theory (e.g., Ellemers and Haslam, 2011; Turner and Reynolds, 2011).

The social identity approach aims to address three core aspects of intergroup behavior: the psychological processes that lead to social identities; the different strategies that people use to derive and maintain a positive social identity; and the key characteristics of the social structure that determine which of these strategies are likely to be used in any given case. The social identity approach posits that a key psychological process underlying group phenomenon is self-categorization: people come to interpret the social world as consisting of *ingroups*—social groups to which the individual belongs—and *outgroups*—social groups of which the individual is not a member (Tajfel et al., 1971; Turner et al., 1987). More specifically, *depersonalization* refers to the psychological process through which people come to perceive the self as an interchangeable exemplar of a social category, rather than a separate individual with unique traits (Tajfel and Turner, 1979; Turner et al., 1987). When individuals categorize themselves as group members, the ingroup becomes integrated with the self and individuals come to recognize the characteristics of the ingroup as representing part of themselves (Smith et al., 1999; Tropp and Wright, 2001). Depersonalization has been argued to be the basis for group cohesion, interpersonal attraction, and social cooperation (Hogg and Turner, 1985). A social identity is integral to an individual’s sense of self, and this self-categorization process has a number of cognitive, affective, and evaluative dimensions that make it such a central part of social life (Ellemers et al., 2002). Social psychological work has demonstrated that compared with those low in identification, individuals high in identification with a group are more likely to think of themselves as ingroup members (e.g., Spears et al., 1997), to feel connected to other ingroup members (e.g., Doosje et al., 1995), to remain committed to their ingroup when faced with threat (e.g., Ellemers et al., 1997), and to be concerned about how their group is treated relative to other groups (e.g., Tropp and Wright, 1999).

The social identity approach holds that identification with one’s group motivates individuals to distinguish their group from others to attain and preserve positive collective self-esteem as a group member (Brewer, 1999). To create and maintain a positive social identity, individuals can adopt one of three main strategies: they can seek to escape, avoid, or deny belonging to a devalued group (*individual mobility*); they can seek to redefine the intergroup comparison by representing the ingroup in terms of positive rather than negative characteristics (*social creativity*); or they can engage in action designed to change the standing of their group (*social competition*). One of the most common ways of preserving a positive social identity comes from the social competition strategy and involves *intergroup bias*: the systematic tendency to evaluate one’s own group or its members more favorably than an outgroup or its members (Tajfel, 1982). The social identity approach suggests that these strategies will be differentially employed based on the extent to which group members perceive the group differences and boundaries to be *permeable, stable, and legitimate* (Tajfel and Turner, 1979). As will be evident in this review, most work considering ingroup favoritism in economic games has focused on the social competition strategy, and often ignored whether group members perceive the social order to be permeable, stable, or legitimate, or whether they engage in the strategies of individual mobility or social creativity.

## Using economic games to explore prosocial behavior

In the social psychological tradition, prosocial behavior refers to the performance of “a broad category of acts that are defined by some significant segment of society and/or one’s social group as generally beneficial to other people” (Penner et al., 2005, p. 366). The most commonly discussed examples of prosocial behavior include helping in emergency situations, volunteering, and donating to charity: i.e., *unidirectional* helping where a helper provides assistance to someone else in need. However, prosocial behavior also includes *bidirectional* social interactions—cooperation—where individuals are aware of the benefits of pursuing the best joint outcomes for all and coordinate their behavior accordingly (Dovidio et al., 2006). Cooperation can be seen as paying a cost to give a (typically greater) benefit to others. In this paper we include both unidirectional helping (e.g., donating to charity) and bidirectional helping (e.g., cooperation in public goods games) under the heading of prosocial behavior: actions that benefit others, often at some immediate cost to oneself. Prosocial behavior has been studied using a range of methods. In this paper we depart from previous reviews in social psychology by focusing our discussion on one specific approach to studying prosocial behavior that has been fruitful in understanding prosocial behavior—namely, the use of economic games.

Game theory—“the study of mathematical models of conflict and cooperation between intelligent rational decision-makers” (Myerson, 1991, p. 1)—provides an important tool for examining human behavior. Game theory is concerned with the decision-making that occurs when one agent interacts with another agent, and can be broadly divided into analytical game theory and behavioral game theory (Camerer, 2003). Analytical game theory often uses mathematical derivations to predict what agents (e.g., people) do in a specific interaction (e.g., an economic game), and is often based on introspection, logic, and mathematical formulae. In contrast, behavioral game theory concerns what players actually do in real situations, and expands upon analytical game theory by also considering the role of emotions, mistakes, doubt, learning, and so on. Behavioral game theory is therefore a branch of economics that explicitly uses psychological regularity (e.g., biases) to extend theories of behavior (Camerer, 2003). With roots in both behavioral game theory and psychology, a number of *behavioral economic games* have been developed and utilized in research. Economic games allow researchers to explore, in tightly controlled experiments, how people make real choices concerning resource distribution. In particular, they are designed with the intent of precisely specifying processes affecting decision making in a way that helps to eliminate any potential external confounding variables, prioritizing tight control of variables over ecological validity. The core feature of economic games is their simplicity, where one player usually has a strictly dominant strategy if he is self-interested, and where this selfish strategy is salient and easy to understand in all cases. If and when a player does not choose this selfish strategy we can infer that they deliberately did not do so—that they had some other motive (Fehr and Schmidt, 2006). One important concept is in economic theory is *utility*, which refers to the perceived ability of something to satisfy needs or wants—that is, to be happiness-producing. Another important concept in game theory is that of the *Nash equilibrium*: a solution concept of a set of strategies in a non-cooperative game whereby each player is assumed to know the equilibrium strategies of the other players, and no player has anything to gain by changing only their own strategy. Put simply, a group of players are in a Nash equilibrium if each one is making the best decision that he or she can, taking into account that the others are also doing their best.

Of special importance to this review are the concepts of preferences and beliefs. These are central components of the behavioral economic approach to social behavior, and this conceptual apparatus provides an effective tool for examining the causal mechanisms behind prosocial behavior. *Preferences* refer to a person’s dispositions towards certain behaviors and outcomes based on the utility expected to be derived from them, while *beliefs* refer to the expectations that people have about uncertain outcomes in a game (Camerer, 2003). Real-world prosocial behavior is subject to a range of psychological influences that make examining the distinct power of preference- and belief-based processes difficult. Consider the example from the Biblical parable of the Good Samaritan, where a man helps a wounded stranger on the road. Did he help because he had a strong motivation to do so? Did he help because he would feel good by doing it? Did he help because he expected some future reward? Did he help because people were watching and he wanted to forge a good reputation? Did he help because he was reluctant to violate social norms that endorse helping others in need? The richness of real-world situations is in part what makes studying prosocial behavior so fascinating, but this very richness also limits the precise delineation of the relative influence of the different contributing psychological processes of preferences and beliefs. In this sense, economic games allow one to strip down situations to more fully examine preferences and beliefs occurring, which then gives greater insight into the real-world phenomenon at hand.

**Figure 1:**
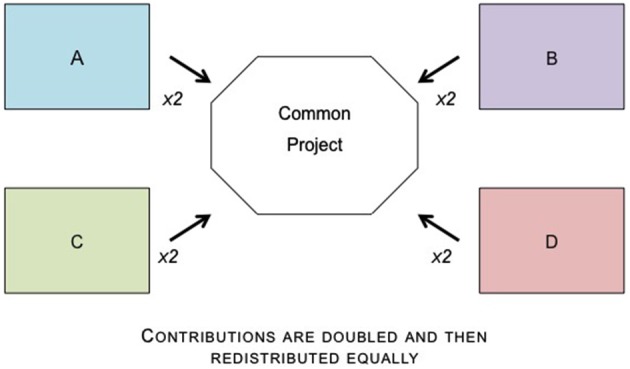
**Public Goods Dilemmas require participants to choose how many points to contribute to a common project.** The points that are contributed are then doubled and then redistributed equally to each player.

**Figure 2:**
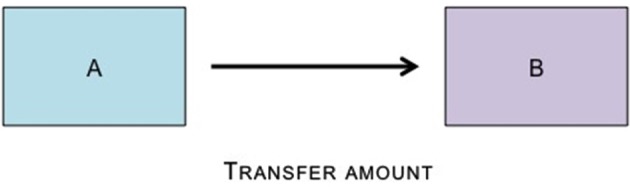
**The Dictator Game has two players.** One player—the dictator—makes a unilateral decision of how to divide an amount of money with a second player—the recipient.

**Figure 3:**
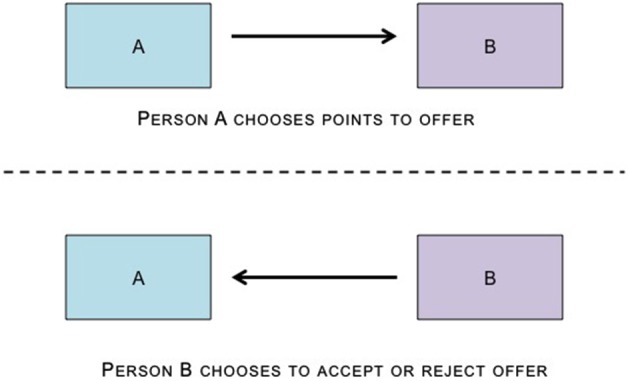
**The Ultimatum Game has two players.** One player (the proposer) receives an amount of money and makes a proposal to the other player (the responder) regarding how to divide the money between them. If the responder accepts the proposed split, both players receive the allocated money. However, unlike the DG, the responder has the option to reject the proposed split, leading both players to receive nothing.

**Figure 4:**
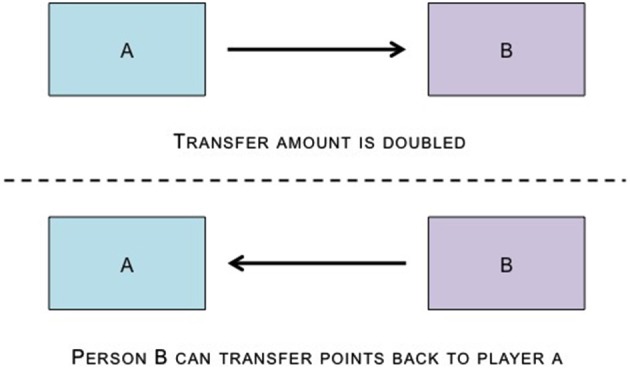
**The Trust Game has two participants, an investor (Person A) and a trustee (Person B).** Person A is given some money and told that they must send a proportion (from zero to the full amount) of this money to Person B, and that the experimenter will multiply the money by some amount. Once Person B receives the money, they are told that they must send back a portion of it to Player A, again ranging from zero to the full amount.

As evident throughout the articles in this special issue, there are a number of economic games used in research. To avoid repetition, in Table 1 we provide a brief summary of the most commonly used games used to study intergroup prosocial behavior and the extent to which they measure preferences and beliefs. Research using economic games to study prosocial behavior has often used a small number of specific games with slight modifications: the *Tajfel Minimal Group Paradigm Matrices* (“Tajfel Matrices”: Tajfel, 1970); the *Prisoner’s Dilemma* (PD; Rapoport and Chammah, 1965; Axelrod, 1980); the *Intergroup Prisoner’s Dilemma* (IPD; Bornstein and Ben-Yossef, 1994); the *Intergroup Prisoner’s Dilemma Maximising Difference* (IPD-MD; Halevy et al., 2008); *Common Pool Dilemmas* (CPD; Hardin, 1968; Messick et al., 1983); *Public Goods Dilemmas* (PGD; Hardin, 1968; see Figure 1); the *Dictator Game* (DG; Kahneman et al., 1986; Forsythe et al., 1994; see Figure 2); the *Ultimatum Game* (UG; Güth et al., 1982: see Figure 3); and the *Trust Game* (TG: Berg et al., 1995; see Figure 4).

**Table 1:** **Commonly used behavioral economic games to study intergroup prosocial behavior**.

Name	Abbreviation	Reference	Brief description	Preferences and beliefs
Common Pool Dilemmas	CPD	Hardin (1968), Messick et al. (1983)	A typical CPD might have four players and a common pool consisting of a certain number of points (e.g., 200 points). In a round, each player can take up to 50 points. This amount is then halved and earned by the player; the remaining money gets split equally among all players.	In a CPD, prosocial behavior can be attributed to some combination of preferences (e.g., a motivation to help the other players) or beliefs (e.g., believing that prosocial behavior will be reciprocated in future).
Dictator Game	DG	Kahneman et al. (1986), Forsythe et al. (1994)	One player—the dictator—makes a unilateral decision about how to divide an amount of money with a second player—the recipient. The dictator is able to allocate any amount of money to the recipient—from nothing to the entire amount—and the recipient must accept this amount.	In principle, the DG excludes any role of beliefs, since the experimental set-up is described in a way that makes it clear that there can be no reciprocity or interdependence of outcomes; the dictator has complete power over the situation and the recipient must accept whatever amount the dictator decides. Therefore, behavior in the DG can be interpreted as resulting primarily from social preferences.
Intergroup Prisoner’s Dilemma	IPD	Bornstein and Ben-Yossef (1994)	In this game there are two groups, with three members in each group. Each player receives an endowment of two monetary units, and is informed that they can either keep this unit or contribute it to a common pool. For every contribution, each ingroup member (including the contributor) gains one unit and each outgroup member loses one unit. Three key strategies can be discerned. First, individuals can play according to the individual strategy, where individually the best strategy is to not contribute anything because the individual’s return from contributing 2 units is only 1 unit. Second, individuals can play according to the ingroup strategy, where the dominant group strategy is for all group members to contribute; this is because group-wide contribution generates a total of 3 units for the ingroup while costing it only 2 units. Third, individuals can play according to the collectively optimal strategy, where because the ingroup’s gain from contribution is exactly offset by the outgroup’s loss, contribution is a net waste of units from the collective point of view, and so the collectively optimal strategy—the one that maximizes the payoff of both groups and all players—is for all players to defect.	Prosocial behavior can be attributed to some combination of preferences (e.g., ingroup love or outgroup derogation) and beliefs (e.g., adherence to social norms of cooperation; expectations of future reciprocity).
				
Intergroup Prisoner’s Dilemma—Maximizing Difference	IPD-MD	Halevy et al. (2008)	In the IPD-MD, group members are able to direct their contributions to one of two pools: a between-group pool, or a within-group pool.The between-group pool parallels the original IPD whereby an increase in the payoffs to each ingroup member by 1 unit decreases the payoff to each outgroup member by 1 unit. In contrast, the within-group pool increases the payoffs to each ingroup member by 1 unit but has no effect on the outgroup. Contribution to the within-group pool indicates ingroup love—the cooperative motivation to increase the ingroup’s payoff. In contrast, contribution to the between-group pool indicates outgroup derogation—the aggressive motivation to hurt the outgroup (or the competitive motivation to increase the ingroup’s relative payoff).	Prosocial behavior can be attributed to some combination of preferences (e.g., ingroup love or outgroup derogation) and beliefs (e.g., adherence to social norms of cooperation; expectations of future reciprocity).
Minimal Group Paradigm Matrices	Tajfel Matrices	Tajfel (1970)	The Tajfel Matrices require individuals to distribute points between other participants who are identifiable only by code number and their group membership (e.g., “participant number 34 from Group A”). Participants are informed that after the task was finished, they will receive the total number of points that had been allocated to them by the other participants. Participants tend to allocate points according to three main strategies: aiming for maximum joint profit of all players, for maximum profit for the ingroup, or for maximum difference in points between the ingroup and outgroup.	Participants cannot allocate points to themselves, which was intended to eliminate direct reciprocity. Therefore, in theory the Tajfel matrices allow the researcher to isolate the contribution of social preferences to ingroup favoritism in economic games.
Prisoner’s Dilemma	PD	Rapoport and Chammah (1965), Axelrod (1980)	In this game, players (the “prisoners”) can choose to cooperate or defect. If players both cooperate, they achieve a good outcome. However, if one player defects while the other cooperates, the defector gets the highest payoff, and the cooperator gets the lowest payoff—giving both players an incentive to defect. If both defect, both do poorly, and so the PD demonstrates the tension that lies between individual rationality (reflected in the incentive of both sides to be selfish) and group rationality (reflected in the higher payoff to both sides for mutual cooperation over mutual defection)	Cooperation can arise from a genuine desire to cooperate with the other player (preferences), the expectation that the other person is likely to cooperate and so it makes sense for you to also cooperate (beliefs), or some combination of the two.
Public Goods Dilemmas	PGD	Hardin (1968)	A typical experimental PGD has four participants who choose how many points to contribute to a common project. The points that are contributed are then multiplied by some amount from 0.25 to 1 and then redistributed equally to each player.	As with other social dilemmas, in a PGD prosocial behavior can be seen to arise from preferences and beliefs.
Trust Game	TG	Berg et al. (1995)	This game has two participants: an investor and a trustee. The investor is given some money and told that they must send a proportion (from zero to the full amount) of this money to the trustee, and that the experimenter will multiply the money by some amount. Once the trustee receives the money, they are told that they must send back a portion of it to the investor, again ranging from zero to the full amount.	With its focus on trust, for the first mover in a one-shot game, behavior is likely to be driven by both preferences and beliefs. In particular, behavior is likely to be motivated by expectations about whether the second player will return any money, and whether this probabilistic outcome justifies the potential gain in winnings. For the second mover in a one-shot trust game, however, behavior is likely to be driven primarily by preferences: does the person want to return some money back to the first player? The trust game, then, taps a mixture of preferences and beliefs.
Ultimatum Game	UG	Güth et al. (1982)	One player (the proposer) receives an amount of money and makes a proposal to the other player (the responder) regarding how to divide the money between them. If the responder accepts the proposed split, both players receive the allocated money. However, unlike the DG, the responder has the option to reject the proposed split, leading both players to receive nothing.	Prosocial behavior by the proposer in the UG can be attributed to a mixture of social preferences and beliefs, as they gauge both how much they would like to offer, but also the likelihood that such an offer would be accepted. Meanwhile, prosocial behavior by the responder, who decides whether to accept or reject the offer, can be interpreted as resulting from preferences alone.

## Preferences and beliefs in prosocial behavior

### Preferences

Social preferences have been incorporated into the behavioral economic approach to account for the finding that individuals do help others even when it is against their interest (Camerer and Fehr, 2004). Put simply, in addition to self-regarding preferences, people have other-regarding social preferences concerning the well being of others, fairness, and reciprocity (Fehr and Schmidt, 1999; Charness and Rabin, 2002; Camerer, 2003).

Social preferences are likely to have been evolutionarily advantageous because cooperation was typically in our long-term best interest. Were we to engage in a cost-benefit analysis of the pros and cons of moving away from a predator every time we encountered one, we would rather quickly end up dead. Rather, it is efficient for humans to have developed intuitive motivations that align with the behavior that is—in general—fitness maximizing. Humans must be equipped biologically to function effectively in many social situations without excessive reliance on cognitive processes, and so pro-social preferences are likely to be part of human nature (Hoffman, 1981; Van Vugt and Van Lange, 2006). It seems likely that some preferences for prosocial behavior—especially towards group members, given our kin-based evolutionary history—have developed because they have been particularly evolutionarily advantageous. Indeed, a growing body of research suggests that people do indeed have prosocial social preferences, with people exhibiting “intuitive prosociality” (Rand et al., 2012; Zaki and Mitchell, 2013).

While slightly different categorizations of social preferences exist in the literature, in this review we follow Fehr and Schmidt (2006), who distinguish three types of other-regarding social preferences (henceforth simply “social preferences” or “preferences”): outcome-dependent; reciprocal; and type-dependent.

#### Outcome dependent social preferences

The simplest kinds of social preferences are *outcome-dependent*, in the sense that they concern only the payoffs to self and others. There are several different types of outcome-dependent social preferences. Positive outcome-dependent social preferences (“altruism”) refer to increases in positive utility for the self associated with gains to others: a person gains satisfaction as another person gains something positive. By contrast, negative outcome-dependent social preferences (“spite”) refer to decreases in positive utility for the self associated with gains to others: a person loses satisfaction as another person gains something positive. *Efficiency* is a preference for outcomes that maximize the sum total of payoffs to all players, regardless of how those payoffs are distributed (Charness and Rabin, 2002; Capraro, 2013). Finally, *inequity aversion* refers to decreases in positive utility for self as material payoffs to others become more inequitable (Fehr and Schmidt, 1999; Bolton and Ockenfels, 2000; Charness and Rabin, 2002). Inequity aversion can take the form of either *advantageous inequity aversion* or *disadvantageous inequity aversion*. Advantageous inequity aversion occurs when an individual gains positive utility from giving up some economic benefit to move in the direction of more equitable outcome: an individual perceives that they have a disproportionately high amount of a resource and desires to redistribute this accordingly. Disadvantageous inequity aversion occurs when an individual experiences negative utility (i.e., dissatisfaction) from their lack of resource compared to another, and gains positive utility with redistribution in the direction of more equitable outcomes.

#### Reciprocal social preferences

The second type of social preferences concerns the fair or unfair behavior of other agents in the game—commonly referred to as *reciprocity*, and exemplified in the Biblical injunction of an “eye for eye” (Leviticus 24:19–21). Here, individual’s positive utility becomes greater as behavior is seen to be reciprocal. Reciprocity refers to the motivation to respond with kindness towards actions perceived to be kind (positive reciprocity) and with unkindness towards actions perceived to be hostile (negative reciprocity). A person’s utility function to help another who has helped them, therefore, will be greater than the desire to help a person who previously has not helped. Such social preferences do not only depend on the allocations of resources, but also on the perceived intentions behind an action (Falk et al., 2003). It is important to note that such reciprocal preferences are distinct from a belief-based process as it is not future material benefits or reward that drive behavior, but rather a preference for reciprocity. Reciprocal preferences occur even in anonymous one-shot economic games where there is no possibility of future reward. This preference-based reciprocity is often referred to as “strong reciprocity”, to be contrasted with the “weak reciprocity” driven by strategic self-interested considerations in repeated interactions (Dufwenberg and Kirchsteiger, 2004; Falk and Fischbacher, 2006).

#### Type-dependent social preferences

The third type of social preferences are *type-dependent*: an individual behaves kindly towards a “good” person with perceived altruistic preferences and with hostility towards a “bad” person with spiteful preferences (Fehr and Schmidt, 2006). Such type-dependent preferences may change an individual’s utility function to engage in prosocial behavior: the perception of the other person as “bad” reduces one’s desire to help them, while the perception of the other person as “good” increases one’s desire to help them. Note that while connected to beliefs (perceptions of character depend on cognitive evaluations about the person and their behavior) such an explanation importantly differs from a belief-based process. For type-dependent social preferences, the suggestion is that the perceived “type” of outgroup members makes people less motivated to act prosocially towards them (preferences), rather than strategically determining that it would be disadvantageous to help them (beliefs).

### Beliefs

In the context of economic games, beliefs are the expectations that people have about uncertain outcomes in a game. While preferences refer to an individual’s own inclinations and desires to act prosocially, beliefs typically focus on the other player and the context in which the game is played. Evidence suggests that strategic beliefs concerning whether cooperative behavior will be reciprocated—and thus whether it is advantageous—are an important driver in observations of prosocial behavior (Fischbacher and Gächter, 2010; Blanco et al., 2014). Such strategic beliefs can come in different forms, which are detailed below.

Overall, the importance of strategic beliefs in explaining prosocial behavior is paralleled by the cost-reward analysis of emergency helping (Piliavin and Piliavin, 1972; Piliavin et al., 1981; Dovidio et al., 1991), which holds that people are motivated to maximize their rewards while minimizing their costs, and so in emergency situations weigh the probable costs and rewards of alternative courses of action before deciding on a decision that will result in the best personal outcome for them.

#### Outcome dependence and direct reciprocity

First, an individual may have beliefs about direct reciprocity and outcome dependance, where the individual is aware that by helping another person, that person is likely to help them back in return, thus being strategically advantageous for both parties. While perceived outcome dependence occurs in some games (e.g., the original Tajfel matrices), many one-shot economic games do not allow for direct reputation building outside the experimental context. In contrast, within social groups, one-shot interactions are rare and interactions occur within a repeated and ongoing context.

#### Reputational concerns and indirect reciprocity

Second, an individual may have beliefs about* indirect reciprocity*: expectations about the likelihood of having prosocial behavior paid back to oneself by another person at a later time (Alexander, 1987; Nowak and Sigmund, 1998, 2005). For example, a person may lend money to a neighbor not due to any preferences to do so, but because they judge it would be strategically advantageous to have a generalized positive reputation amongst their neighbors because at some point they are likely to need help from a neighbor themselves. Evolutionary accounts positing a reputation-based theory of cooperation in groups argue that through being helpful in situations where others know that the helper will not benefit directly, the person builds a reputation of being someone trustworthy, thus enhancing evolutionary fitness. Milinski et al. found that when reputation building was allowed for in a public goods paradigm, participants cooperated more and so were more productive: “the “tragedy of the commons” was no longer a tragedy; instead, the commons became productive and could be harvested” (Milinski et al., 2002, p. 426). Helping in an economic game is affected both by the image score of the recipient and the image score of the donor: that is, donors with higher image scores help more, particularly for recipients who also have good reputations (Seinen and Schram, 2006). Similarly, Croson (2007) has conducted a set of social dilemma experiments to test between the commitment, social preferences, and reciprocity explanations of prosocial behavior, and found strong support for reciprocity over the other two theories. Indeed, some work has suggested that in PGDs, cooperation can be utilized as a reputational strategy and that the benefits of the action to the society at large can sometimes be of secondary importance (Van Vugt and Hardy, 2010). Indirect reciprocity—reputation building—is an important factor in explaining prosocial behavior in economic games (Rabin, 1993; Levine, 1998; Dufwenberg and Kirchsteiger, 2004).

#### Cooperative norm violation

Thirdly, strategic beliefs can be connected to social norms, whereby people act more prosocially towards group members because they perceive that to be the socially approved form of action and are aware of the costs of violating such norms (Fehr and Fischbacher, 2004a).

## Ingroup favoritism

Having outlined the conceptual framework of preferences and beliefs, we now describe in detail how this framework can help to elucidate the phenomenon of ingroup favoritism. A number of experimental studies, using adults in both real and artificial groups in economic games, have found that *intragroup* prosocial behavior is consistently higher than *intergroup* prosocial behavior: i.e., people consistently act more prosocially towards ingroup members than outgroup members. Social identity appears to influence prosocial behavior even in young children: 3–7 year old children exhibit greater generosity towards ingroup members than outgroup members across a series of economic games (Fehr et al., 2008), and 6 year old children punish third-party selfishness more harshly when it comes from an outgroup member and when it disadvantages ingroup members (Jordan et al., 2014b). It has been shown that the presence of a subtle cue of relatedness facilitates group cooperation in a PGD (Krupp et al., 2008) and that highlighting relatedness promotes general prosocial motives and behavior in a DG-like charitable donation game (Pavey et al., 2011). Experimentally making social identity salient leads to ingroup favoritism in the UG, for both the responder and proposer roles (McLeish and Oxoby, 2011), and white responders higher on implicit racial bias discriminate against Black individuals in an UG, accepting more offers from other White participants than Black participants, even at a cost to their own financial gain (Kubota et al., 2013). Even artificially created minimal groups exhibit ingroup favoritism: in the PD (Ahmed, 2007); the Tajfel Matrices (Tajfel, 1970); the IPD-MD (Halevy et al., 2012); and PGDs and CPDs (Kramer and Brewer, 1984; Brewer and Kramer, 1986). Such findings have also been observed in the field—in a DG in post-conflict Bosnia (Whitt and Wilson, 2007), and in PGDs in both homogenous and heterogeneous communities in Uganda (Habyarimana et al., 2007). Finally, this pattern is even exhibited in non-human primates: capuchin monkeys, for example, have been observed to act prosocially selectively towards ingroup members in a simple resource distribution task (de Waal et al., 2008).

Much empirical research demonstrating ingroup favoritism has been conducted using the principle of recategorization (Gaertner et al., 1993): if group membership boundaries are modified, do our parochial boundaries of prosocial behavior change? The creation of a common ingroup identity has been shown to lead to increased helping behavior (Nier et al., 2001) and support for more cooperative intergroup policies (Beaton et al., 2008). Using economic games, Kramer and Brewer (1984) conducted three CPD experiments to assess the effects of making salient either a superordinate or subordinate group identity and found that individuals were most cooperative when a superordinate identity was salient. Such results were found again by the same researchers using a PGD (Brewer and Kramer, 1986), and have received support by a number of other researchers using similar manipulations. Using artificial experimental groups, de Cremer and Stouten (2003) have found that once people develop a common social identity this shared identity leads to more trust and cooperation in a PGD, and selfish individuals in a public goods game can be encouraged to cooperate by increasing the salience of their common ingroup identity (de Cremer and Van Vugt, 1999). In further support, Wit and Kerr (2002) have found that members of artificial groups exhibited greater cooperation in social dilemma games upon recategorization to a collective common ingroup identity. Finally, such principles have been demonstrated in real groups by Rand et al. (2009).

Across multiple games, by a number of different researchers, using different groups and populations, the behavioral observation of ingroup favoritism has been well documented. Our prosocial tendencies, in other words, are parochial. Why might this ingroup favoritism occur?

## Preferences in ingroup favoritism

With regards to ingroup favoritism, preference-based accounts of ingroup favoritism cohere around the notion that group membership alters an individual’s social *preferences* in economic games: intergroup processes change people’s desires and inclinations concerning the outcomes of ingroup and outgroup members. In economic terms, identity influences an individual’s utility function (Akerlof and Kranton, 2000). In this section we discuss how group membership may relate to the three types of preference-based accounts distinguished above: positive and negative social preferences over others’ outcomes, reciprocity, and type-dependent preferences (Fehr and Schmidt, 2006). In general, preference-based accounts of ingroup favoritism align well with the social identity approach.

### Outcome-dependent social preferences

#### Ingroup love

One of the most prominent preference-based explanations of ingroup favoritism in social psychology is rooted in work from the social identity approach and posits that people simply have a stronger desire—i.e., a social preference—to help ingroup members relative to outgroup members. To use Brewer (1999) terminology, individuals exhibit greater prosocial behavior towards other ingroup members relative to outgroup members in economic games because they show *ingroup love*, brought about by categorization processes of *depersonalization*. On the ingroup love account, identifying with a group leads to ingroup favoritism because the individual’s own interests become more aligned with the interests of the group collective, thus enhancing the desire to help others—just as one would want to help oneself (Tajfel and Turner, 1979; Brewer and Kramer, 1986; Turner et al., 1987). Simpson (2006) provides evidence that ingroup favoritism stems from an increase in how actors weigh the payoffs to fellow ingroup members rather than through changes in expectations about fellow ingroup members’ actions.

One way of empirically testing the ingroup love hypothesis comes from work on relative group identification. If ingroup favoritism arises through depersonalization-based social preferences where the ingroup is included within the self, individuals who identify more strongly with their group should also be those that act more prosocially towards ingroup members. Indeed, greater attachment to a group—and so presumably greater depersonalization—has been shown to be associated with greater cooperation in a PGD (de Cremer, 2002). When taking part in a PGD in which cooperation is breaking down, high group identifiers given an attractive exit option to leave the game exhibit a significantly greater desire to remain in the group (to restore cooperation), even when this is against their own economic best interests (Van Vugt and Hart, 2004). It seems that because high group identifiers had a stronger sense of we-ness that triggered greater concern for the outcomes of other members of the group too, they were more likely to remain cooperative.

Further support is found in research on empathy, the complex and multifaceted ability to share the emotional states of others (Preston and de Waal, 2002; de Waal, 2008). The empathy-altruism hypothesis explicitly explains prosocial behavior in terms of empathy-based altruistic preferences, where the perception of another person’s need in conjunction with a special interpersonal relationship to that person (based on, for example, group membership) evokes empathy, which in turn increases altruistic motivations (e.g., Batson, 1991; Batson and Shaw, 1991). Accordingly, empathic social preferences are known to have general prosocial effects in economic games. For example, empathy sustains cooperation in a PGD (Rumble et al., 2010), and participants induced to feel empathic concern in a PD tend to show higher levels of cooperative responses (Batson and Moran, 1999)—even when they know that their partner has already made a competitive choice (Batson and Ahmad, 2001). Work using neuroscientific methods has provided further empirical support for this by demonstrating an *intergroup empathy bias*: the tendency to empathize more with ingroup relative to outgroup members, (Cikara et al., 2014). Studies using functional magnetic imaging (fMRI) report that people show more neural activation in pain and empathy circuits when observing the pain of an ingroup member, compared to an outgroup member (Xu et al., 2009; Chiao and Mathur, 2010; Gutsell and Inzlicht, 2010, 2012; Cheon et al., 2011). Such findings support the claim that the interests of ingroup members have become adopted—to a degree—as the interests of the self. In an intergroup setting, Mathur et al. have demonstrated both that the neural responses within regions associated with empathy are heightened for the pain of ingroup relative to outgroup members and further that this relative neural activity predicts altruistic motivation for one’s ingroup in the form of paying more to help ingroup members (Mathur et al., 2010). Of particular importance is work from Hein et al., who investigated the neural processes that preceded the willingness to engage in costly helping toward ingroup and outgroup members. Participants were able to choose to help an ingroup or outgroup member by enduring physical pain themselves to reduce the other’s pain. Hein et al. found that helping the ingroup member was best predicted by anterior insula activity—an area known to be critical for empathy. In contrast, lack of help to the outgroup member was best predicted by nucleus accumbens (NAcc) activity—an area associated with reward processing and aggression (Hein et al., 2010). It seems, then, that ingroup-favoring preferences are an important driver of the effects of social identity on prosocial behavior in economic games: social identification increases prosocial behavioral by reducing actors’ tendency to draw distinctions between their own and others’ welfare, such that a member of a group may get positive utility when the total welfare of his group increases.

#### Outgroup derogation

The counterpart to the positive social preference of ingroup love is the negative social preference for *outgroup derogation*, or* outgroup hate* (Hewstone et al., 2002). It is known that in addition to positive social preferences for prosocial behavior, some individuals exhibit spite, having social preferences for negative outcomes of others—for example, punishing prosocial behavior in others (Anderson and Putterman, 2006). To what extent can outgroup derogation explain ingroup favoritism in economic games?

Neuroimaging and behavioral data suggests that in addition to positive social preferences for ingroup members, people may experience pleasure in response to out-group members’ adversities (*Schadenfreude*) and displeasure in response to their triumphs (*Glückschmerz*, Leach et al., 2003; Smith et al., 2009; Cikara et al., 2011). Indeed, on both theoretical and empirical grounds, such negative counter-empathic responses are distinct from ingroup love-based ingroup empathy (Cikara et al., 2014). With regards to empathic responses, it has been argued that the intergroup empathy bias may be better explained by negative counter-empathic responses towards outgroups, rather than ingroup love (Cikara et al., 2014). An individual, therefore, may lose positive utility when the total welfare of an outgroup increases.

Alongside this, however, a growing body of research suggests that, by and large, ingroup love may be a more potent driver of intergroup relations than outgroup derogation (Mummendey and Otten, 1998; Hewstone et al., 2002). Of particular importance is work by Halevy et al. (2012), who explored the role of preference-based ingroup love and outgroup derogation in explaining ingroup favoritism using the IPD-MD. They found that group members were not competitive or aggressive *per se*, and that when given the choice participants strongly preferred to cooperate to maximize their absolute group gains, rather than compete against the outgroup for relative gain. Further, their results revealed that ingroup love prevails over outgroup derogation even after intergroup conflict (Halevy et al., 2012). Research using alternative PD matrices has shown that ingroup favoritism arises even in the absence of an outgroup and intergroup comparison, suggesting that ingroup love is more potent in driving in ingroup favoritism than outgroup derogation (Gaertner et al., 2006). Most recently, meta-analytic findings from Balliet et al. (2014) show that the effects of ingroup love are stronger than outgroup derogation in explaining ingroup favoritism.

Overall, evidence suggests that both positive and negative social preferences concerning outcomes play a role in leading to ingroup favoritism—but that positive social preferences for ingroup love may play a stronger role than negative social preferences for outgroup derogation.

#### Inequity aversion

Advantageous inequity aversion may be more prevalent in exchanges with ingroup members due to a desire to minimize within-group differences (Turner et al., 1987). When engaging in exchanges with outgroup members, however, a competitive desire to maximize ingroup payoffs relative to the outgroup may predominate over inequity-based feelings of guilt for outgroup members. Advantageous inequity aversion, therefore, might be moderated by group membership such that increased guilt is felt when a group member has a disproportionate share of resources compared to other group members. Evidence for such group-moderated advantageous inequity aversion was found by Chen and Li (2009), who used a series of simple economic games including the DG and Tajfel Matrices, and found that participants gave 47% more in charitable redistributions to ingroup relative to outgroup members. Similarly, participants experienced less disadvantageous inequity aversion when an ingroup member (compared with an outgroup member) received a higher payoff than they themselves did. Chen and Li’s findings suggest that both advantageous and disadvantageous inequity aversion play a role in ingroup favoritism.

### Reciprocity

The second type of social preferences concerns the fair or unfair *behavior* of other agents in a game, where an individual’s positive utility becomes greater as behavior is seen to be more reciprocal. Positive reciprocity appears to be moderated by group membership, with Chen and Li (2009) finding that group members were 19% more likely to respond prosocially to (that is, reward) an ingroup member for good behavior compared to an outgroup member. While research on this is relatively scarce, it seems that social preferences for positive reciprocity may be more pronounced among group members, such that ingroup members are rewarded more for prosocial behavior than outgroup members.

Group membership also seems to be an important factor in negative reciprocity. One well-studied form of negative reciprocity is altruistic punishment—the social preference to punish those who violate the norms of cooperation, even when it is implausible to expect that these costs will be repaid by others or at a later date (Boyd et al., 2003). Altruistic punishment constitutes a social preference because of people’s tendency to act in this way even outside of any clear benefit to themselves, with people often punishing those who violate norms of cooperation towards a third party (Fehr and Fischbacher, 2004a,b; Jordan et al., 2014a).

Initial evidence suggested that individuals may exhibit less altruistic punishment towards ingroup members (Bernhard et al., 2006). It seems that the extension of positive regard to ingroup members may lead individuals to punish ingroup defectors more leniently due to greater feelings of warmth towards them. This has also been explored through examination of *third-party punishment*: third parties who observe a norm violation are willing to incur costs to punish the norm violator (Fehr and Gächter, 2002; Fehr and Fischbacher, 2004b). In a group context, Bernhard et al. (2006) looked at group membership and third party punishment in real groups in Papua New Guinea and found that third parties gave more lenient judgements to ingroup norm violators than outgroup norm violators. Similarly, further recent work has found that ingroup members are punished less than outgroup members for misbehavior (Chen and Li, 2009; Mussweiler and Ockenfels, 2013). Most recently, it has been found that children punish outgroup members more harshly than ingroup members (Jordan et al., 2014b). Such results can be taken as indicating a preference-based account whereby group membership may moderate the use of altruistic punishment towards ingroup members.

However, other work has suggested that group membership moderates negative reciprocity preferences and altruistic punishment in the opposite direction, where ingroup members may be punished more for selfish behavior than outgroup members. Goette et al. compared randomly assigned minimal groups to randomly assigned real groups and found that real groups punished ingroup defectors significantly more harshly than did minimal groups (Goette et al., 2006). Similarly, it has been found that participants who are cooperative in a gift-giving game punish noncooperative ingroup members more severely than they punish noncooperative outgroup members (Shinada et al., 2004). Most recently, Mendoza et al. (2014) had participants play as recipients in an UG, finding that participants exacted stricter costly punishment on racial ingroup members than outgroup members (Study 1). In a second study they replicated this effect of greater costly punishment towards college ingroup members rather than outgroup members, and further found that such punishment was magnified among strong college group identifiers. Finally, a third study found evidence suggesting that such group-moderated costly punishment was driven by violated expectations of fairness from ingroup members. It is therefore unclear exactly how group membership moderates negative reciprocal preferences in intergroup contexts, and future work is necessary to elucidate the conditions under which ingroup members are punished to a greater or lesser degree.

### Type-dependent social preferences

Perceived immorality seems to be particularly relevant to intergroup type-dependent preferences. It is known that intergroup conflict is characterized by a sense of moral superiority: “we” are more honest, peaceful, trustworthy, and friendly than “they” (Brewer, 1999). As the ingroup becomes larger, older, and more established, the norms, rules, and institutions that maintain cooperation often take on the character of moral authority, which can lead to emotions of denigration and contempt towards outgroup members (Brewer, 1999). Ingroup favoritism in economic games could therefore be explained in part due to type-dependent preferences regarding morality. Ingroup members are perceived to be more moral, which increases the motivation to help them, while outgroup members are perceived to be more immoral, affecting a person’s preference to engage in prosocial behavior, leading to reduced prosocial behavior.

Related to perceived immorality, outgroup members may also be perceived as more threatening which is also likely to negatively impact upon social preferences. Indeed, a primary source of negative affect towards outgroups results from threat of perceived incompatibility of their goals with ingroup goals (Fiske and Ruscher, 1993). Work suggests that group membership can moderate the perception of immoral others: immoral outgroup members are seen as posing a *realistic threat* to one’s resources and safety, while immoral ingroup members are seen as posing *a symbolic threat* to the group’s image and reputation (Marques et al., 1988; Branscombe et al., 1999). In an intergroup context, it is known that group members report less desire to interact with targets depicted as lacking moral qualities, and that this is mediated for outgroup members by perceptions of threat (Brambilla et al., 2013). Given the central role that perceptions of threat play in intergroup conflict (see LeVine and Campbell, 1972; and Sherif (1966) Realistic Conflict Theory), it is likely that perceptions of threat to material resources and safety will impact negatively upon prosocial behavior in economic games, with players being selectively prosocial towards ingroup members because they are seen as less threatening.

Therefore, in addition to simple outcome-dependent preferences and preferences for reciprocity, it is possible that type-dependent social preferences concerning the perceived character of other players also play a role in explaining prosocial behavior in intergroup contexts. While this has received little attention in economic games compared to other types of preference-based explanations reviewed here, it seems plausible that ingroup favoritism in economic games may in part result from the perception of outgroup members as immoral, and particularly as posing a realistic threat to one’s own resources.

## Beliefs in ingroup favoritism

### Interdependence of outcomes and direct reciprocity

One influential belief-based account suggests that ingroup favoritism in economic games can be explained as a result of perceived outcome interdependence and expectations of reciprocity. An influential early criticism of the ingroup love account of ingroup favoritism in the Tajfel matrices was proposed by Rabbie et al., who argued that ingroup favoritism in point allocation was the result of beliefs about *outcome interdependence* rather than social preferences (Rabbie et al., 1989). That is, participants implicitly perceive their own outcomes to be dependent on their choices: “by giving more to their ingroup members than to the outgroup members—in the expectation that the other ingroup member will reciprocate this implicit cooperative interaction—they will increase their chances of maximizing their own outcomes” (Rabbie et al., 1989, p. 176). Indeed, work conducted in the years after Tajfel’s initial studies documented that while social categorization can be a sufficient condition for intergroup discrimination, this is by no means a universal response, and can be extinguished by feedback concerning how other members of the ingroup and outgroup respond (Locksley et al., 1980). That is, learning that outgroup members do not discriminate against one’s own group, and learning that one’s own group does not discriminate against the outgroup, both help to reduce discrimination. Beliefs concerning the behavior of other group members are evidently of great importance. According to Rabbie et al., participants make ingroup-favoring allocations due to perceived outcome interdependence: even if participants cannot allocate money to themselves, they can give to others who may in turn reciprocate their allocations. Rabbie et al. suggest that participants perceive stronger outcome dependance with ingroup members, which in turn leads to higher expectations of reciprocal behavior by ingroup members. It is this, they argue, that leads participants to allocate more points to ingroup members. Rabbie et al. concluded that allocations in the Tajfel matrices were not motivated by ingroup love but rather reciprocal expectations: the *reciprocity hypothesis* (Rabbie et al., 1989).

Scholars have distinguished between two versions of such a theory: a strong* (unbounded)* version and a weak (*bounded*) version. In the strong unbounded version, participants attempt to maximize their outcomes by allocating more resources to others upon whom they perceive themselves to depend for their own outcomes, anticipating that this favorable treatment will be returned. This strong, unbounded, version makes no claim concerning group membership: this can occur for both ingroup and outgroup members (Stroebe et al., 2005). Since individuals are more likely to engage in repeated interactions with someone from their own social group, the weaker version—the “*bounded reciprocity hypothesis”*—suggests that the effects of reciprocity are bounded, or constrained, by social categorization (Gaertner and Insko, 2000). On this account, beliefs about reciprocity are influenced by both group membership and interdependence, such that people have higher expectations of reciprocity from ingroup members, and this leads to ingroup favoritism (Locksley et al., 1980). Applied to the minimal group studies with which it was formulated, the bounded reciprocity hypothesis suggests that participants who are dependent on their ingroup for their own outcomes have stronger ingroup reciprocal expectations and consequently make more ingroup-favoring allocations in the Tajfel matrices than participants who are not. In contrast, outgroup outcome dependance will not lead to outgroup reciprocal expectations or to outgroup-favoring allocations (Stroebe et al., 2005). Group membership, in other words, moderates the effects of interdependence and reciprocity in the Tajfel matrices.

In a series of experiments using artificial groups, Yamagishi et al. have provided evidence suggesting that ingroup favoritism in economic games occurs only when participants believe other ingroup members will reciprocate the favor. Initial evidence for this was provided when Yamagishi et al. conducted a partial replication of Tajfel’s minimal group experiments using the Tajfel matrices (Karp et al., 1993). In their study, they had two conditions: a replication of the original minimal group studies where the participant making the allocation decision was also subject to allocation decisions by other members; and a modified condition where allocators were told they would be paid a fixed amount of money and that this would not depend upon others’ allocation decisions. Their results revealed that ingroup favoring decisions emerged when the subject was also a target of allocation decisions by others, but not when subjects’ payoffs did not depend on allocation decisions by others. Such results speak against an (ingroup love) preference-based account, which would predict ingroup favoring decisions whether or not the participant could receive allocation rewards from others or not. In contrast, such results support the role of a distinct belief-based channel: that people only show ingroup favoring decisions when they expect the favor to be reciprocated. In a later study, it was found that participants only acted more cooperatively in a PD when they believed that the recipient had knowledge that the participant was an ingroup member, consistent with the notion that cooperation was driven by the expectations of ingroup reciprocity (Yamagishi and Kiyonari, 2000). This is further supported by work from Gaertner and Insko (2000), who had participants allocate rewards in a minimal group paradigm but varied whether the other allocator was an ingroup or outgroup member, and whether participants would personally receive rewards or not. They found that participants favored the ingroup over the outgroup, but only when they were dependent on an ingroup member for their own outcomes. Similarly, Stroebe orthogonally manipulated participants’ dependance on an ingroup and an outgroup member for outcome rewards in a modified Tajfel Matrices task and found again that ingroup favoring strategies were strongest with (but not exclusive to) outcome dependance on the ingroup (Stroebe et al., 2005).

Work on expectations of reciprocity and interdependence, then, supports a model where individuals respond to the dependance structure and then reciprocate with favoritism towards those on whom they are dependent, with this effect considerably stronger for the ingroup (hence “bounded”) due to the perception of the group as a “container of generalized reciprocity” (Yamagishi and Kiyonari, 2000; Stroebe et al., 2005). Indeed, a recent meta-analysis of cooperation in ingroup favoritism has provided substantial support for this (Balliet et al., 2014), with situations involving interdependence of outcomes resulting in stronger ingroup favoritism in social dilemmas (*d* = 0.42) compared to the weaker (but still significant) effect of games with no interdependence of outcomes (e.g., DG; *d* = 0.19). It is clear, however, that while important, interdependence of outcomes cannot completely explain ingroup favoritism, for such behavior is observed even in non-interdependent games. Why might this be so?

### Indirect reciprocity and reputational concerns

Reputational concerns can lead to ingroup favoritism if group members are strategically concerned with signaling a positive reputation towards other ingroup members. That is, individuals may believe that it is advantageous to selectively act prosocially towards ingroup members when they can build a reputation of being a prosocial person. In line with this, in more recent years Yamagishi et al. have expanded on their earlier work that primarily concerned the potential for direct reciprocity (Jin and Yamagishi, 1997; Yamagishi et al., 1998, 1999), to put forward their BGR Model. They suggest that the presence of a salient ingroup activates a default group heuristic strategy, which explains the higher incidence of prosocial behavior within groups (Yamagishi and Kiyonari, 2000; Yamagishi and Mifune, 2008, 2009). The BGR model has three core ideas: first, that humans evolved to have depersonalized and generalized trust that other ingroup members will cooperate; second, that people are motivated to establish and maintain a cooperative reputation among ingroup members because of the strategic advantages this brings; and third, that people expect to be the beneficiaries of prosocial behavior from other ingroup members, but not necessarily from the same ingroup members they cooperated with or helped previously (Kiyonari and Yamagishi, 2004). On the BGR account, people treat ingroup members more favorably than outgroup members not because of ingroup love but because they anticipate favorable treatment from ingroup members through both direct and indirect reciprocity (Yamagishi and Kiyonari, 2000). Such beliefs concerning expectations of reciprocity can be connected to stereotypes: group members believe that others in their group are likely to act prosocially towards them, which influences behavior. Awareness of these generalized norms of reciprocity within groups, Yamagishi et al. argue, accounts for observations of ingroup favoritism, particularly in social dilemmas where one’s own payoff depends on the actions of other group members. Note that for this account, the focus is on the expectations of future rewards, rather than (as in the preference-based accounts) a motivation to respond like-with-like.

Evidence for this comes from work by Yamagishi and Mifune, who found that participants only allocated more money to ingroup members in a DG when they believed that others knew their own group membership (Yamagishi and Mifune, 2008). Similarly, ingroup favoritism in a DG occurs only when participants are exposed to reputation monitoring cues, suggesting that it is adherence to norms of reciprocity and beliefs about indirect reciprocity rather than ingroup love that drives cooperative behavior within groups (Mifune et al., 2010). They argue, therefore, that, such beliefs are necessary for ingroup favoritism to occur in minimal groups: that “ingroup favoritism does not occur when participants cannot or do not expect favorable treatments from ingroup members” (Yamagishi and Kiyonari, 2000, p. 127).

An important limitation of this current work on bounded reciprocity is that it has largely focused on ingroup favoritism in artificial groups. Will real groups exhibit the same behavior in economic games as artificial groups? Individuals often attach much importance to their social groups in real life, and cooperation is commonly found in groups whose members interact on a regular basis, have emotional ties to one another, share a common frame of reference, and are behaviorally interdependent—conditions absent from minimal group paradigms (Tajfel and Turner, 1979; Levine and Moreland, 1994). While a minor form of this ingroup attachment can be generated in minimal group paradigms, it remains clear that these minimal group memberships may not generate sufficient attachment and emotional involvement to consistently affect preference- or belief-based prosocial behavior in artificial experiments. Indeed, people in self-selected real groups are more cooperative in simultaneous dilemmas than were their counterparts in minimal groups, and this effect is mediated by group identity (Jackson, 2008). Such results are consistent with previous findings linking group identification to cooperative responses to a social dilemma (Kramer and Brewer, 1984; de Cremer and Van Vugt, 1999). Recent work looking at beliefs in ingroup favoritism has suggested that ingroup favoritism can be explained by both beliefs and preferences: using real university groups in a DG, participants transferred more to ingroup members than outgroup members, but that this was particularly so when the recipient knew the group membership of the participant (Ockenfels and Werner, 2014). It is clear that further research is needed to investigate the effectiveness of the BGR model in predicting prosocial behavior in real groups, where social identity processes are likely to be more important.

### Cooperative norm violation

A final belief-based explanation of group behavior in economic games concerns social norms, whereby people act more prosocially towards group members because they perceive that to be the socially approved form of action and are aware of the costs of violating such norms (Fehr and Fischbacher, 2004a). It is known that identifying with a group enhances adherence to group norms (Terry and Hogg, 1996; Jetten et al., 1997), and that group beliefs typically involve an injunctive norm of cooperation: prosocial cooperative behavior toward ingroup members is expected (Tajfel and Turner, 1979). Identification with a group not only increases the likelihood that one will follow group norms, but also leads people to anticipate that other group members are likely to follow such group norms (Terry and Hogg, 1996; Mullin and Hogg, 1998), and indeed cooperative responses in social dilemmas are more common if cooperative responses by other players are also expected (Messick and Brewer, 1983; Seinen and Schram, 2006). In addition to a possible preference-based process where an individual internalizes cooperative norms and acts prosocially accordingly (Kerr et al., 1997), awareness of these norms and the costs of breaking them may also lead to prosocial behavior via a strategic belief-based process.

The anticipation that others will also follow cooperative group norms is likely to bolster an individual’s own intention to follow the social norm of cooperation lest they be seen as a deviant group member and incur the associated costs. Social norms are often infused with a moral dimension, and violations of these norms are often accompanied by punishment from others within the group (Cialdini and Trost, 1998). In other words, the costs of acting selfishly in interactions with ingroup members may be greater than the costs of selfish behavior with outgroup members due to the existence of cooperative norms within groups. Some evidence suggests that deviant group members are punished more than deviant outgroup members. For example, as discussed above with regard to altruistic preferences, research has shown that participants who are cooperative in a gift-giving game punish noncooperative ingroup members more severely than they punish noncooperative outgroup members (Shinada et al., 2004), and participants playing as recipients in an UG exact stricter costly punishment on racial ingroup members than outgroup members (Mendoza et al., 2014). Such findings can be taken to support both preference-based accounts concerning reciprocity, and cooperative norm violation. As we shall repeat later in this review, the two processes are not mutually exclusive.

Participants’ awareness of the costs of violating ingroup cooperative norms may increase the likelihood of within-group prosocial behavior. Support for this comes from Habyarimana et al. (2007), who conducted a series of PGDs in Uganda where participants sometimes played with ingroup members (“co-ethnics”) and sometimes outgroup members (“non co-ethnics”). In addition to finding evidence of ingroup favoritism, they present results suggesting that such behavior can be explained through a belief-based process whereby ingroup members cooperate because they adhere to within-group norms, believing in the power of sanctioning those who violate such norms.

## Preferences and beliefs: separation and integration

In this paper we have reviewed how decades of work on ingroup favoritism has detailed experimental evidence strongly implicating distinct roles for preferences and beliefs. Ingroup favoritism is multiply determined: both preferences and beliefs are important, and both play a role. The challenge now is to explore these processes further, both in separation and in integration.

Theoretical and empirical separation of these processes is essential in research seeking to explain ingroup favoritism, and an important limitation of some commonly used economic games is that they are often unable to isolate preferences and beliefs. For example, in a PGD any observed prosocial cooperation could be explained in terms of preferences (ingroup love; inequity aversion) or beliefs (adherence to norm of reciprocity; reputational concerns), or some combination thereof. Given this, researchers should attempt to complement the use of these measures with those that provide a tighter way to isolate the individual channels of preferences and beliefs. For example, to explore social preferences researchers could utilize decision problems with a single decision maker who is tasked with distributing resources between a group of people that includes both ingroup and outgroup members. Another approach could be to compare one-shot vs. iterated games, or compare anonymous with public games. Such theoretical and empirical separation of preferences and beliefs is necessary to allow a better understanding of how, why, and when group processes affect prosocial behavior.

At this juncture, the reader may question which of these are more critical: are preferences or beliefs principal in explaining ingroup favoritism? This is a natural question, but one that is impossible to answer at present based on existing research. While meta-analytic work by Balliet et al. (2014) has suggested a stronger role for beliefs than preferences in ingroup favoritism, it is also likely that in some circumstances preferences will be more powerful. Moreover, in everyday situations both preferences and beliefs work together as mutually enforcing. It is likely that an individual with greater social preferences to help the ingroup will also have stronger beliefs that facilitate this, just as individuals with stronger social identification to a group also exhibit greater belief in and adherence to the norms of that group (Terry and Hogg, 1996; Jetten et al., 1997). As Fehr and Schmidt (2006) argue, it is likely that prevailing social norms affect participants’ preferences, such that long-standing social practices are internalized and directly affect social preferences. For example, stereotypic expectations (beliefs) about a certain group being immoral are likely to reduce the motivation to act prosocially towards them (preferences), such that the resulting ingroup favoritism is a function of a form of preferences over beliefs, where beliefs directly influence preferences.

We suggest that group members are likely to show the greatest ingroup favoritism in economic games where both preferences and beliefs are able to influence behavior. Ingroup favoritism is most likely to occur within real groups that the participant has emotional attachment to (preferences for) and where the situation involves injunctive cooperative norms towards ingroup members, interdependence of outcomes, and expectations of reciprocity from others (beliefs). Such a situation can be seen as characterizing the “optimal” conditions in which ingroup favoritism will occur. In contrast, eliminating some of these channels (for example, using a minimal group, or by excluding reputation building by making interactions anonymous) will decrease the frequency of prosocial behavior. For example, we would predict that ingroup favoritism would be stronger in interdependent social dilemmas than in DG, and particularly so when decisions are public and the groups used are real-life groups that the individual strongly identifies with. In contrast, it is likely that ingroup favoritism would be at its weakest in DG (with no interdependence of outcomes) played with minimal groups and where decisions are private.

We further suggest that these factors can interact such that preferences and beliefs reinforce each other and lead to greater prosocial behavior than might be predicted from the mere sum of their parts. Indeed, theoretical work on *psychological game theory* has suggested that in addition to simple preferences and beliefs, in some cases—for example, guilt-, individual’s preferences can depend on beliefs (Battigalli and Dufwenberg, 2007, 2009). For example, if Jones transfers less to Smith in a game than Smith was expecting, Jones may feel guilty for letting Smith down (preferences), but the extent of the guilt that Jones feels is likely to also depend on how much Jones thinks that Smith thinks that Jones let him down (beliefs). In this case, preferences and beliefs are mutually reinforcing. Similarly, evidence suggests that beliefs about the expectations of others are related to their type-dependent preferences (Aguiar et al., 2009, Brañas-Garza and Rodriguez-Lara, unpublished manuscript). With regards to ingroup favoritism, it is possible that upon self-categorization as a member of a group, depersonalization leads to ingroup love, which may simultaneously reduce the greed motive in social dilemmas by changing the focus from self-interest to group-interest (preferences), while also establishing a role for beliefs concerning direct reciprocity and interdependence of outcomes and the need to preserve a positive reputation (beliefs). As these beliefs are internalized, social identification with the group may become greater, further increasing the likelihood of ingroup favoritism. It would be fruitful for future work to directly examine this hypothesis.

## Future directions: an integrative approach

Considering how preferences and beliefs shape ingroup favoritism facilitates a more complete understanding of ingroup favoritism. This division of preferences and beliefs is certainly not new to social psychology, having echoes in the debate regarding ingroup favoring tendencies in the minimal group paradigm studies of the 1970s. We believe, however, that the explicit delineation of preferences and beliefs in ingroup favoritism provides new insights for future work in both social psychology and behavioral economics. In this section we describe three such integrative approaches. First, we describe how social psychology has highlighted that not all groups are alike, and that the specific content of beliefs (stereotypes) about groups and the affective motivations (preferences) they engender differ across groups, which is likely to influence prosocial behavior. Second, we suggest that ingroup favoritism may be explained in part through considerations of group reputation: a form of preferences over beliefs. Third, we discuss how work on ingroup favoritism can begin to incorporate other key insights from the social identity approach, over and above social competition as intergroup bias. Overall, we show how integration of theories from social psychology with the preferences and beliefs framework provides important new directions for researchers investigating prosocial behavior in groups.

### The diversity of groups

Behavioral economic research has tended to assume that outgroups are perceived in the same (negative) way. But are all outgroups the same? Social psychological work suggests not. If we consider the range of groups about which we hold certain beliefs, a vast array of beliefs can be observed: this group is mean, another kind; that group in need of help, another a threat; and so on. The Stereotype Content Model (Fiske et al., 2002) holds that such stereotype content is captured by two dimensions: *warmth*—linked to perceptions of common or competitive goals—and *competence*—related to the target group’s overall status in society. In their model, specific groups are associated with different patterns of warmth and competence. For subordinate, non-competitive groups (e.g., the elderly), the positive stereotype of high warmth interacts with the negative stereotype of low competence to maintain the advantage of more privileged groups (a “paternalistic” stereotype). In contrast, for high status, competitive groups (e.g., Jews) the positive stereotype of their competence justifies the overall system but acts jointly with negative stereotype of low warmth to justify overall resentment (an “envious” stereotype). In the behavioral economic lexicon, individuals have different beliefs and expectations about the likely behavior of distinct groups.

What about preferences? The stereotype content model has been extended by the BIAS Map (Behaviors from Intergroup Affect and Stereotypes) (Cuddy et al., 2007), which proposes that stereotypes lead to emotions, which then lead to behaviors of discrimination and conflict. Warmth stereotypes determine active behavioral tendencies, increasing or decreasing motivations for active harm (harassing) and eliciting active facilitation (helping), while competence stereotypes determine passive behavioral tendencies, attenuating passive harm (neglecting) and eliciting passive facilitation (associating). Such work highlights that preferences (the emotions and motivations deriving from stereotypes) and beliefs (the stereotypical expectations of behavior from group members) are likely to interact in predicting prosocial behavior, with each informing the other.

An informative direction for future research, then, would be to explore the way in which beliefs and preferences associated with different groups interact to elicit ingroup favoritism. There has been a tendency in work conducted using a behavioral economic methodology to explore ingroup favoritism towards “generic” outgroups, and yet a wealth of research from social psychology has highlighted that not all outgroups are perceived alike. Such differential perceptions are likely to impact upon the likelihood of ingroup favoritism being observed. Based on work from the stereotype content model and the BIAS map (Fiske et al., 2002; Cuddy et al., 2007), it is likely that ingroup favoritism is likely to be more pronounced towards outgroups subject to an envious stereotype, because such beliefs evoke feelings of threat, defensiveness, and resentment. In contrast, ingroup favoritism may be weaker in interactions with outgroups subject to a paternalistic stereotype, due to the beliefs that such people are kind but non-threatening. Such work would help to inform our understanding of the psychological processes underlying ingroup favoritism through demonstrating the way in which preferences and beliefs interact in different contexts.

### Group reputation

Do individuals care about their *group reputation*, as well as their own strategic individual reputation? We argue that they do, and that this can be explained as a function of integrated preferences and beliefs. As we have discussed, much work conducted in behavioral economics and evolutionary biology has argued that individuals may act in a prosocial way selectively to ingroup members as a way of signaling that they have a good character and resources to help others—that is, that they have good evolutionary fitness and should be considered potential social partners (Zahavi, 1975; Alexander, 1987; Nowak and Sigmund, 1998, 2005). This suggests that beliefs may drive this effect, through individuals expecting that the probabilistic outcome of interacting with the person that they helped at some point in the future is significantly higher for ingroup members than outgroup members. Aside from the expected tangible benefits (e.g., receiving help in the future, or evolutionary success), is this all that there is to reputational concerns? We argue that there is more, and do so through integrating preferences with beliefs. Reputation management occurs when a person cares about another’s beliefs about some unobservable attribute of theirs and then takes into account how their choices affect others’ beliefs. What does “caring” mean? It could be interpreted as “caring” in the sense of holding beliefs that it would be advantageous for the other person to hold positive beliefs about you. On the other hand, it could also implicate a role for preferences: aside from probabilistic benefits, people might gain some intrinsic benefit from having a positive reputation. We suggest it can refer to both.

How might preferences connect to group reputational concerns? Social psychological work in the social identity approach suggests that through depersonalization, individuals come to take on the interests of the group as self-interests (Tajfel and Turner, 1979; Turner et al., 1987; Brewer, 1999). Indeed, a key part of the social identity approach is that individuals are motivated to preserve a positive social identity, both to themselves and others. This, we argue, is connected to reputational concerns: group members often act in ways that will preserve the positive reputation of the group. On such a preference-based account, individuals genuinely care about both the material outcomes of the group as well as its reputational identity. Combining these theoretical perspectives, we therefore suggest that in addition to being concerned about their individual reputations, people will also act in ways to preserve a positive group reputation, even when there are no clear material gains to be expected in the future.^1^ We call this the *group reputation hypothesis*.

What evidence is there for the claim that through social identity processes, individuals may seek to maintain a positive group reputation? Particularly important is work on *strategic helping*: the claim that group members are motivated to signal a positive identity for their group and that they believe prosocial behavior can be an effective way of doing this (e.g., Nadler and Halabi, 2006; Hopkins et al., 2007). Social psychological work on helping in intergroup contexts has highlighted the strategic role that such helping can have: prosocial behavior plays an important role in social identity creation and maintenance, and groups create, maintain, or challenge status relations through helping (Nadler, 2002; Nadler and Halabi, 2006; Hopkins et al., 2007; Nadler et al., 2009). Just as helping at the interpersonal level can serve as reputation management (Nowak and Sigmund, 1998, 2005; Milinski et al., 2002), prosocial behavior has also been posited as reputation management at the group level (Hopkins et al., 2007). That is, group members may seek to perform actions that they believe will induce outgroup members to see ingroup members in the positive way that ingroup members see them.

While intergroup signaling can lead to ingroup favoritism, it is particularly interesting because it can also lead to the reverse: preferential helping behavior towards outgroup members. When individuals are motivated to signal the prosociality and kindness of their group, prosocial behavior may actually be extended more towards outgroup members. Hopkins et al. (2007) experimentally tested the role of intergroup signaling in prosocial behavior using the known stereotype of Scots as mean. After establishing that Scots do indeed believe they are seen as mean by the English and resent this stereotype, Hopkins found that Scots believed that outgroup helping was a particularly effective way of refuting this stereotype. It was found that increasing the salience of the stereotype of Scots as mean resulted in an increase in help volunteered to outgroup—but not ingroup—members (Hopkins et al., 2007). Such results highlight that helping others may be a means to advance a group’s interest through social competition, and so intergroup signaling can lead to group moderated prosocial behavior—though this time outgroup favoring, rather than ingroup favoring. Indeed, the potential for evaluation by another group can cause group members to act more prosocially (van Leeuwen and Oostenbrink, 2005) and groups may actually compete to act more positively towards the other and consequently gain a positive group reputation (Jetten et al., 1996). As discussed above, in the Stereotype Content Model, group stereotypes are organized along dimensions of both warmth and competence (Fiske et al., 2002). Prosocial behavior between groups serves as an effective way of signaling to others that one’s own group is both warm (i.e., moral, caring, friendly) and competent (i.e., having both resources and ability), and so may be particularly well suited as a form of reputation-management action. Such work suggests that through depersonalization, individuals come to value a positive group identity for its own sake (social preferences), as well as anticipating that prosocial action is a way of achieving this, and that positive reputations have strategic advantages (beliefs).

Our integrative approach shows how combining the concepts of preferences and beliefs from behavioral economics with the body of work conducted in social psychology on social identity processes can lead to novel research directions. By and large, behavioral economics has largely considered beliefs about reputations at the individual level: an individual signaling to another individual(s) that they are a good potential exchange partner. At present it has neglected, however, work suggesting that individuals also care about the reputation of their group (distinct from their individual reputation) and will act in ways to create and maintain a positive group identity through prosocial behavior. Social psychological work highlights that in addition to individual-level signaling, people also engage in group-level signaling. On the other hand, work on the social identity approach has often neglected the strategic role of signaling a positive reputation, often conceptualizing it as a function of ingroup love rather than its potential as a way of influencing outcomes in future. It would therefore be fruitful for future research to examine processes of group reputation signaling in economic games.

### Social identity strategies

Reflecting the state of the literature, most of the work reviewed here has focused on the role of ingroup favoritism as a form of group competition—intergroup bias—whereby people acting more prosocially towards ingroup members provides objective benefits to other ingroup members that (may) give them an advantage compared to another group. Yet, as discussed earlier, the social identity approach consists of far more than just intergroup bias—it is a grand, overarching theory that considers not just the individuals within a group but the social structure within which group relations are explored (Ellemers and Haslam, 2011). In particular, the social identity approach is often misunderstood as suggesting that identification with a group will always lead to intergroup bias where the ingroup is inevitably favored over the outgroup—yet this is false (Ellemers and Haslam, 2011). Rather, the social identity approach requires appreciation of the different identity strategies (individual mobility; social creativity; social competition) that individuals may pursue to achieve a positive social identity, as well as the specific structural conditions relating to both how the group boundaries are perceived and the objective possibility of change (permeability; legitimacy; stability) (Tajfel and Turner, 1979; Turner et al., 1987; Turner and Reynolds, 2011).

In this sense, research purporting to support other theories of ingroup favoritism—e.g., the BGR model—perhaps should not be taken to simultaneously provide evidence against a social identity account. Such alternative findings are compelling, and yet the extent to which they speak against a social identity account is limited for the very reason that the “social identity” account such results are compared against should best be considered a form of “social-identity-lite”. That is, social identity theory offers much more insight into intergroup relations than the mistaken claim that identification with a group will always lead to intergroup bias where the ingroup is inevitably favored over the outgroup (Ellemers and Haslam, 2011). Social identity theory can explain not just the diversity of groups, but also the ways in which ingroup favoritism may or may not be manifested depending on the structural characteristics of the intergroup context and the groups involved.

Greater consideration of the social structure and context of the groups will help to provide a fuller understanding of the topic at hand. To frame the issue in the behavioral economic lexicon, what role do preferences and beliefs play in leading an individual to adopt a particular identity strategy, and how might the social structure impact upon one’s preferences and beliefs? It is not our place here to present an entire proposal of research connecting the social identity approach and ingroup favoritism, though we do think that there are a number of places in which it may be a good part to start.

One feature of the social identity approach that is clearly limited in current research concerns the use of different identity strategies in leading to ingroup favoritism in economic games. For example, is it inevitable that individuals will always be more altruistic towards ingroup members in these games? We suggest that in some situations it is likely that individuals may also employ the other strategies: social creativity and individual mobility. How might maximization-based preferences (e.g., ingroup love) interact with intragroup inequity-aversion when in competition with each other? Would individuals prefer their group to have more resources overall, or for individuals in the group to have equal resources? Existing research suggests that in a neutral setting in which no prescriptive norm is externally imposed, welfare maximization is a stronger motivation than inequity aversion (Charness and Rabin, 2002; Engelmann and Strobel, 2004; Capraro et al., 2014), yet it remains unclear how this might be manifested in intergroup contexts.

To what extent do people feel that they can leave their group, and consequently engage in an individual mobility strategy rather than a social competition-based ingroup favoring resource allocation strategy? For example, some work has highlighted that individuals will leave their group as a response to the free rider solution (Yamagishi, 1988), suggesting that in some cases individuals may be likely to engage in an individual mobility strategy over social competition. For example, a weakly identified non-religious (but ethnic) Jew may choose to leave the group, rather than engage in ingroup favoritism against a more dominant group. The use of this strategy is likely to be moderated by the extent to which individuals can leave their group, and the prior commitment and identification they feel towards the group. To explore this, research might compare cases in which group boundaries are relatively stable and legitimate (e.g., British undergraduates, who apply for and enter their degree course to study a specific subject), compared to those which are less so (e.g., American undergraduates, who have flexibility to choose and switch majors during the start of their course).

As another example, an important question for future research could be to ask when might people seek to cognitively reinterpret the giving situation, perhaps redefining selfish actions as being better than ingroup favoritism? Are group members more likely to exhibit ingroup favoritism when they perceive the group boundaries as stable and legitimate? Research might explore this through comparing ingroup favoritism in groups that individuals elected to join (e.g., university; sports club) with those in which they had no choice (e.g., parental income; ethnic background).

How might the status of the groups concerned impact upon this behavior—for example, might high status groups exhibit less ingroup favoritism towards low status groups than vice versa? Research could explore this in a minimal group paradigm through assigning the different groups varying levels of starting monetary units, to create higher and lower status groups. One might expect that low status groups exhibit greater ingroup favoritism than high status groups.

While research has tended to neglect some of the core features of the social identity approach, it does not necessarily need to do so. In fact, considering the complete social identity approach will help to shed light on a number of questions concerning ingroup favoritism, thus being of benefit to researchers from all disciplines working in this area. For this reason, greater consideration of the social identity approach has the potential to be a useful lens through which behavioral economists can examine research questions, just as the behavioral economic approach provides a useful toolbox for social psychologists.

## Conclusion

In this review, we have demonstrated the importance of disentangling preferences and beliefs in explaining ingroup favoritism. We have shown how behavioral economic methods provide a solid conceptual framework through which to examine intergroup prosocial behavior, while also showing that this perspective can be importantly enriched through consideration of social psychological research and theory. We hope that through this integrative review we inspire researchers working in behavioral economics to make use of theory from social psychology, while also inspiring social psychologists to make use of behavioral economic methods.

We end by noting that it would be amiss to consider the issue of ingroup favoritism as just a game that scientists play (pun intended), with merely theoretical and methodological implications of interest just to other scientists. Rather, this issue is one that has far-reaching implications for society at large. The ability of *homo sapiens* to cooperate and act prosocially is undoubtedly one of the biggest—if the not *the* biggest—reasons why our species has flourished. A physically rather weak species, we have colonized the earth through cooperation and team work: the development of liberal democracies, the invention of the computer and the creation of life-saving drugs all arise from the ability of our species to work together. Despite this, however, it is clear that our cooperative tendencies still leave a lot to be desired. In a globalized world, our parochially altruistic tendencies with their roots in our ancestral past in the African savannah are no longer adequate. To ensure the survival of our species in the face of drastic and rapid climate change, poverty, population increases, famine, war, and disease we need now—more than ever—to work together across nations, ethnic groups, religious groups, and so on. Psychology has a very real and important role to play in exploring how we can encourage widespread cooperation across group lines. As Martin Luther King, Jr said, “We must learn to live together as brothers or perish together as fools.” (Speech in St. Louis, Missouri, March 22, 1964: King, 1992).

## Conflict of interest statement

The authors declare that the research was conducted in the absence of any commercial or financial relationships that could be construed as a potential conflict of interest.

## Footnotes

^1^It could be argued that such group-level reputation becomes a public good, such that contributing to a positive group reputation creates a social dilemma. On the other hand, such group reputation could be inextricably linked to personal reputation, if by deviating from the positive group reputation one is actually seen as less of a member of that group. Regardless, it remains an interesting question as to exactly how individuals psychologically navigate group reputation in intergroup prosocial behavior.
